# The Osteogenic Potential of Mesoporous Bioglasses/Silk and Non-Mesoporous Bioglasses/Silk Scaffolds in Ovariectomized Rats: *In vitro* and *In vivo* Evaluation

**DOI:** 10.1371/journal.pone.0081014

**Published:** 2013-11-12

**Authors:** Ning Cheng, Yuanqin Wang, Yufeng Zhang, Bin Shi

**Affiliations:** The State Key Laboratory Breeding Base of Basic Science of Stomatology (Hubei-MOST) & Key Laboratory of Oral Biomedicine Ministry of Education, School & Hospital of Stomatology, Wuhan University, Wuhan, People’s Republic of China.; University of Southern California, United States of America

## Abstract

Silk-based scaffolds have been introduced to bone tissue regeneration for years, however, their local therapeutic efficency in bone metabolic disease condition has been seldom reported. According to our previous report, mesoporous bioactive glass (MBG)/silk scaffolds exhibits superior *in vitro* bioactivity and *in vivo* osteogenic properties compared to non-mesoporous bioactive glass (BG)/silk scaffolds, but no information could be found about their efficiency in osteoporotic (OVX) environment. This study investigated a biomaterial-based approach for improving MSCs behavior in vitro, and accelerating OVX defect healing by using 3D BG/silk and MBG/silk scaffolds, and pure silk scaffolds as control. The results of SEM, CCK-8 assay and quantitative ALP activity showed that MBG/silk scaffolds can improve attachment, proliferation and osteogenic differentiation of both O-MSCs and sham control. *In vivo* therapeutic efficiency was evaluated by μCT analysis, hematoxylin and eosin staining, safranin O staining and tartrate-resistant acid phosphatase, indicating accelerated bone formation with compatible scaffold degradation and reduced osteoclastic response of defect healing in OVX rats after 2 and 4 weeks treatment, with a rank order of MBG/silk > BG/silk > silk group. Immunohistochemical markers of COL I, OPN, BSP and OCN also revealed that MBG/silk scaffolds can better induce accelerated collagen and non-collagen matrix production. The findings of this study suggest that MBG/silk scaffolds provide a better environment for cell attachment, proliferation and differentiation, and act as potential substitute for treating local osteoporotic defects.

## Introduction

Postmenopausal osteoporosis is a kind of bone metabolic disease induced by estrogen deficiency. It is characterized by disequilibrium of bone formation and microarchitecture deterioration, leading to enhanced bone fragility and a consequent increased risk of fracture[1]. OVX animal model is widely recognized to closely represent the pathophysiological situations of postmenopausal osteoporosis. Drilled-hole defect at femural epimetaphyseal is an easy-handling and highly reproducible method, and is a favorable model to test biofunctional properties of scaffolds and to investigate cancellous bone regeneration, especially in load-bearing long bone[2,3]. There is no necessity for additional fixation and doesn’t cause a high risk of post-surgery fracture, as compared to other fracture defect models. Recently, osteoporotic femur defect was used to evaluate the therapeutic effect of biomaterials or drug delivery in skeletal metabolic diseased environment, along with the advantages of easy handling, reliable surgical reproducibility and free to mechanical interference[4,5].

Bioactive glasses (BGs), referred to as the third generation of biomaterials for in situ tissue regeneration, have the capability to bond to both bone and soft tissue[6]. The release of Na^+^ and Ca^2+^ ions and the deposition of a carbonated hydroxyapatite layer form a strong chemical bond between glass and host bone, thus stimulate new bone growth[7]. Additionally, a new member of bioactive glasses mesoporous bioglasses (MBGs), elicit more superior bioactivity than BGs, owing to the highly improved surface and porous volume. MBGs have an ordered mesopore channel structure with a pore size ranging from 5 to 20 nm[8]. Both intracellular and extracellular responses were rapidly improved at the interface of the glass that release soluble Si, Ca, P and Na ions, which promote their novel application in biomedical science[9,10]. However, the inherent brittleness of ceramic scaffolds could produce a non-continuous and collapsed pore network with reduced mechanical strength, which limits their application especially in bone regeneration[11,12]. 

Silk fibroin is a potent biopolymer as bone graft material, primarily due to several favorable properties such as optimal biocompatibility and controllable degradation rates with low immunogenic and inflammatory response[13,14]. In addition, silk possesses β-sheet-rich protein structure, leading to the excellent mechanical characteristics such as stiffness and toughness values superior to other natural and synthetic polymers. Furthermore, silk fibroin has been fabricated into electrospun fibers, thin films, hydrogels, three dimensional porous scaffolds, microspheres and composite materials for tissue engineering and controlled release of drugs[15–19]. Based on the above characteristics and the ability to be processed into a wide range of material formats, silk is a promising candidate material for bone tissue applications. 

In our previous study, we have reinforced silk fibroin matrix with 10 wt. % BGs or MBGs filler to create protein-ceramic composites through a freeze drying method[20]. 3D porous composite scaffolds were synthesized by combining the bioactivity and osteoconductivity of bioglasses and ideal mechanical strength of silk. We found that MBG/silk scaffolds have greater physiochemical properties (mechanical strength, in vitro apatite mineralization, Si ion release and pH stability) compared to BG/silk scaffolds. However, the therapeutic effects of BG/silk and MBG/silk scaffolds in osteoporotic model have been so far negligibly studied. Thus, this report focuses on evaluating the osteogenic potential of silk-based scaffolds on MSCs and femur repair for osteoporotic critical sized defects, investigating the involved bone remodeling process and the therapeutic effect based on a tissue engineering approach. We hypothesize that MSCs have the best property of proliferation and osteogenic differentiation on MBG/silk scaffolds compared to pure silk and BG/silk scaffolds, and the local transplantation of MBG/silk scaffolds would accelerate and best efficiently improve the quality of new bone formation during osteoporotic defect healing. 

## Materials and Methods

### 1: Ethics Statement

The surgical procedures and the guidelines for animal care were approved by the Ethics Committee at the School of Dentistry in Wuhan University (Permit Number: 2011011), People’s Republic of China. Female Wistar rats weighing 200-220g were used for this experiment, and all animals were kept at room temperature under a 12-hour light/dark cycle with food and water ad libitum. All operations were carried out under sterile conditions with a gentle surgical technique. The surgeon was blinded to the treatment. The rats were subjected to bilateral ovariectomy (OVX) or sham operation (Sham) at age of 3 months, according to our previous reports[21]. 

### 2: Mesenchymal stem cells isolation, culture and osteogenic induction

Mesenchymal stem cells (MSCs) from both OVX and Sham rats were isolated from after two months induction. The cell isolation and culture protocol was followed according to the report by Maniatopoulos et al [22]. All femurs and tibia were cut at the epiphysis level, and the bone marrow is flushed out by injecting αMEM with a 10ml syringe. Cells were harvested and washed with Hanks’ balanced salt solution (HBSS), plated to 10cm dishes and cultured in αMEM (Hyclone, USA) containing 10% FBS (GIBCO, Australia) and 1% antibiotic/antimycotic (Hyclone, USA). After 48h incubation at 37°C in an atmosphere of 5% CO_2_, the medium was changed to remove non-adherent cells and renewed every three days. When 80% confluence was reached, the attached MSCs cells were digested with 0.25% trypsin/EDTA (Hyclone, USA) and replated for further expansion. After three passages, cells were used in the following experiment.

OVX MSCs (O-MSCs) were counted and seeded at 1×10^5^ cells per well of a 24-well plate, Sham MSCs (S-MSCs) were also seeded in the same condition as control. Osteogenic differentiation was evaluated by culturing cells with osteogenic media consisting DMEM (Hyclone, USA) supplemented with 10% FBS, 10mM sodium β-glycerol phosphate, 50 μg/ml L-ascorbic acid and 10^-8^M dexamethasone (Sigma-Aldrich, USA) for 14 d and analyzed with Alizarin Red-S staining. 1g Alizarin powder (Sigma-Aldrich, USA) was dissolved into 50ml distilled water, and the staining PH=4.2 was maintained by adding ammonium hydroxide solution. Cells were fixed with 10% paraformaldehyde in PBS for 20 min at room temperature and rinsed in distilled water, followed by staining in Alizarin Red-S solution for 15 min and washed in distilled water by shaking to remove excess stain. The calcium content was quantified by eluting the staining with 10% cetylpyridinium chloride and measuring the absorbance of supernatants at 550nm. All cell assays were done at least in triplicate.

### 3: Cell Seeding and proliferation on 3D scaffolds

3D Silk-based scaffolds (silk, BG/silk, MBG/silk) were investigated with respect to O-MSC/S-MSC proliferation using a Cell-Counting Kit (CCK)-8 (Dojindo, Kumamoto, Japan). Initially, 3×10^4^ cells were suspended in a total of 25ul medium for efficient seeding on 5×5×1mm scaffolds (n=4 per group) without spillage of cells. After 2h cell attachment, prewetted scaffolds were transferred to 48-well culture plates (Corning, USA) in DMEM culture supplemented with 10% FBS at 37°C and 5% CO_2_. The medium was renewed every three days for 1, 3, 7, 11 and 15 d. At each time point, CCK-8 assay was monitored by adding 20ul of CCK-8 solution reagent to each scaffold with a total of 200ul medium and incubated at 37°C. After 2h incubation, 100ul of incubated cell suspension from each scaffold was transferred to a 96-well plate, and the absorbance of supernatants was read at 450nm on a plate reader. 

### 4: Scanning electron microscopy (SEM)

For cellular morphology observation, O-MSC/S-MSC cultured scaffolds were fixed in 4% paraformaldehyde for 1h before the removal of paraformaldehyde solution, and samples were washed twice with PBS, dehydrated in a graded series of ethanol for 10 min each, and followed by CO2 critical-point drying method. Then the specimens were coated with gold and observed under SEM (Vega-3, Tescan, Czech Republic).

### 5: Alkaline phosphatase assay

2×10^5^ cells were seeded on 8×8×2mm scaffolds (n=3 per group) with osteogenic media in a 24-well plate. Quantitative alkaline phosphatase (ALP) activity was measured at 7 and 14 d. At each time point, the culture media was removed and the scaffolds were rinsed with PBS three times, then cells were treated with 0.1% Triton X-100 and 2mM MgCl_2_ for protein extraction. Cell suspensions were transferred to 1.5ml tubes and then were centrifuged at 14,000 rpm at 4°C for 15min. The ALP activity and total amount of protein were determined by *p*-nitrophenyl phosphate method [23]. 

### 6: Femur defect drilling and implantation

Two months after induction, the OVX rats were randomly allocated into four groups (n=6 per group) for bilateral femur drilling: 1).Drill-control group; 2). Silk group; 3).BG/silk group; and 4).MBG/silk group. Before implantation, these scaffolds were sterilized by ethylene oxide. A linear skin incision of approximately 1 cm in the distal femoral epimetaphyseal was made bilaterally and blunt dissection of the muscles was performed to expose the femoral condyle. Briefly, a 2.5-mm-diameter latero-lateral bicortical channel was created approximately beneath the growth plate and perpendicular to the shaft axis, by using a trephine bur at a slow speed irrigated under saline solution to avoid thermal necrosis. The drilled holes were rinsed by injection with saline solution in order to remove bone fragments from the cavity. Implant scaffolds were then gently placed to fill the drilled defects according to group allocation. Subsequently, the incision was closed as mentioned above. Postoperative penicillinum (40,000 IU/ml) was intramuscularly administrated for 3 days. No perioperation or postoperation fractures were produced. At time points, 2 and 4 weeks after scaffold insertion, rats were sacrificed and specimens were harvested for the following evaluation, accordingly.

### 7: μCT analysis

All femurs were placed in a custom-made holder with 4% paraformaldehyde and scanned by a *μ*CT imaging system (*μ*CT50, Scanco Medical, Basersdorf, Switzerland) in a direction perpendicular to the long axis of the drilled channel. Scanning was performed at 55 kV and 114 µA with a thickness of 0.048 mm per slice in medium-resolution mode, 1024 reconstruction matrix, and 200 ms integration time. A Gaussian filter (sigma = 0.8 and support = 1) was used to remove noise. The mineralized bone tissue was differentially segmented to exclude the non-mineralized tissue with a fixed threshold (value = 190). To identify the establishment of osteoporosis model, sections in distal femoral head and femoral shaft were selected both from OVX rats and Sham rats at two months post ovariectomy, and a series of slices starting at a distance of 1 mm proximal from the end of the growth plate with a length of 2 mm were chosen for evaluation. To analyze the bone regeneration process within the defect, the central 1.5-mm-diameter region of the 2.5-mm-diameter circular defect was included as area of measurement per slice, thus to exclude the native bone margins. 100 slices per sample were included to obtain a consistent VOI. After 3D reconstruction, new bone formation was evaluated by using a protocol provided by the manufacturer of the micro-CT scanner. The following parameters were automatically determined: the bone volume faction (BV/TV), the mean trabecular number (Tb.N), the mean trabecular thickness (Tb.Th) and the mean trabecular separation (Tb.Sp). All digitalized data and 3D images were generated by the built-in software of the µCT. 

### 8: Histological staining and analysis

Samples were then decalcified in 10% ethylene diaminetetraacetic acid (EDTA) for 2 weeks, changed twice per week. Samples were dehydrated in graded ethanol from 80% to 100% and embedded in paraffin. Longitudinal serial sections of 5 μm were cut and mounted on polylysine-coated microscope slides. Hematoxylin and eosin (H&E) staining and Safranin O staining (Sigma #S2255; Sigma-Aldrich, St. Louis, USA.) were performed for general histological evaluation according to manufacturer's protocol. Polarized light microscopy (Olympus BX 60, Japan) was used to observe the alignment and orientation of regenerative bone lamellar structure within defect region using H&E slides. 

Bone regeneration of these histological sections was scored on a semiquantitative scale by an individual blinded observer to compare the bone regeneration ability of our scaffolds, using a modified scoring method of Osathanon [24] (Table **1**): (0). no bone formation; (1) minimal bone formation; (2) low bone formation (≤1/4 of the defect); (3) moderate bone formation (≥1/4 and ≤1/2 of the defect); (4) abundant bone formation (≥1/2 and ≤3/4 of the defect); (5) extensive bone formation (≥3/4 of the defect). By using Image J 1.44 software from the National Institute of Health (NIH, Bethesda, Maryland, USA), the fraction of scaffold remnants was calculated as previously described [25,26]. Tartrate-resistant acid phosphatase (TRAP) staining (Sigma #387A; Sigma-Aldrich, St. Louis, USA.) was performed to detect the existence and amount of osteoclasts during 4 weeks healing, indicating the level of bone resorption and scaffolds degradation. According to a previous report, the number of osteoclasts was counted under a light microscope (Olympus DP71, Japan) [27]. The TRAP-positive cells with more than three nuclei were defined and counted as osteoclasts. The bone histomorphometry and TRAP-positive multinuclear osteoclast measurements within defect region were performed on four consecutive ×100-sections per sample in each group at each time point per analysis. From each section, three randomly selected fields (1024×1536 pix) were identified and averaged.

**Table 1:** Semi-quantitative scale for evaluation of bone regeneration.

Score	Extent of new bone in defect
0	No bone formation
1	Minimal bone formation (only very small portion in the defect)
2	Low bone formation (less than one-fourth of the defect)
3	Moderate bone formation (less than one-half and more than one-fourth of the defect)
4	Abundant bone formation (less than three quarter and more than one-half of the defect)
5	Extensive bone formation (more than three quarter of the defect)

### 9: Immunohistochemical study

Immunohistochemical assessment was conducted to determine the expression of osteogenic markers such as COL1, osteopontin (OPN), bone sialoprotein (BSP) and OCN, according to the following procedure. Deparaffinised sections were first incubated in 0.3% hydrogen perioxide in PBS at room temperature for 20 min to block endogenous peroxidase activity, followed by incubation with 5% bovine serum albumin (BSA) at 37°C. The following primary mouse monoclonal antibodies (Abs) were used: anti-BSP (1:100; Boster Biological Technology, Ltd, China), anti-COL1 (1:100; Boster Biological Technology, Ltd, China), anti-OPN (1:200; Biomedical Technologies, Stoughton, MA) and anti-OCN (1:100; Boster Biological Technology, Ltd, China). Tissue sections were incubated with primary Abs in a humidified chamber overnight at 4°C. Sections incubated with PBS instead of primary Ab were used as negative control. After three times washing with PBS, sections were incubated with biotinylated secondary antibody (Zhongshan Biotechnology Co., Ltd, China) for 20 min before incubation with horseradish peroxidase-conjugated avidin-biotin complex (ABC) (Zhongshan Biotechnology Co., Ltd, China) for another 20 min, followed by buffered 3,3-diaminobenzidiinetetrahydrochloride (DAB) (Zhongshan Biotechnology Co., Ltd, China) as chromogen. Sections were then counterstained with hematoxylin, dehydrated with ascending concentrations of ethanol solutions, xylene, and mounted with coverslips. 

### 10: Statistical analysis

All statistical analysis was performed by using SPSS 17.0 software (SPSS, Chicago, IL). Data were expressed as mean±standard deviation (SD) and were analyzed using one-way ANOVA and t-test. For the bone regeneration score, the Kruskal-Wallis H test was used followed by Mann-Whitney U tests if statistically significant. A 5% (P < 0.05) level of significance was adopted.

## Results

### 1: Reduced osteogenic potential in osteoporosis model

**Figure 1:**
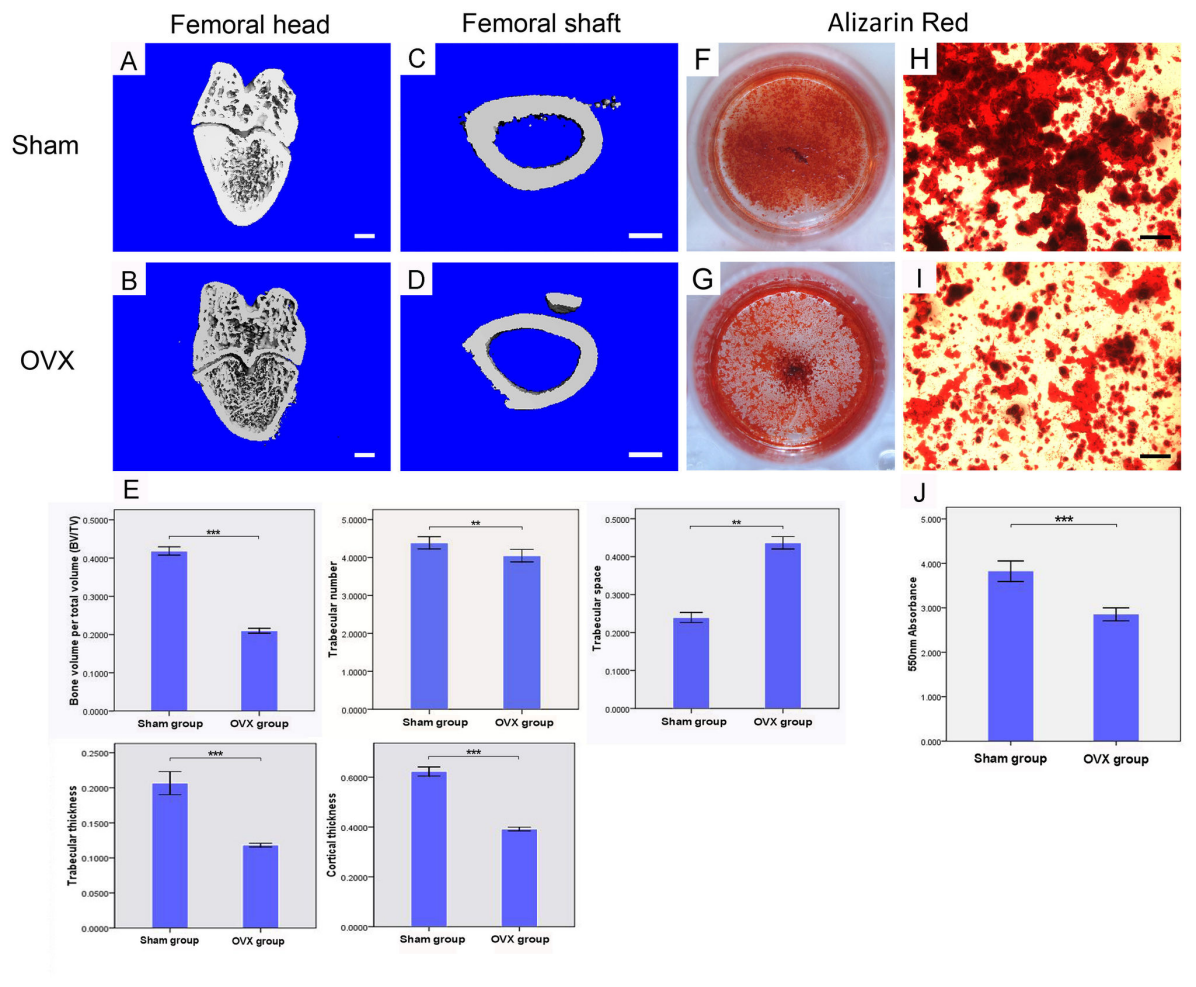
Establishment of OVX model. 3D-μCT images of distal femoral head (A) and femoral shaft (C) in Sham rats; 3D-μCT images of distal femoral head (B) and femoral shaft (D) in OVX rats. Quantitative data of BV/TV, Tb.N (trabecular number), Tb.Sp (trabecular space), Tb.Th (trabecular thickness) in femoral heads and Cor. Th (cortical thickness) in femoral shafts between OVX and Sham rats (n=6 per group). (E; Bar=1mm) Alizarin Red staining for Sham (F,H) and OVX (G,I) rats derived MSCs culture in osteogenic medium at day 28. Quantitative comparision of mineralized nodules between Sham and OVX MSCs (J). Cell assays were performed in triplicate. (Bar=100µm) ** P<0.01, ***P<0.001.

All animals healed uneventfully after surgical procedures. Compared with Sham control, μCT images from OVX rats showed a significant decrease in the trabecular bone volume and microstructure, and reduced cortical thickness with expanded marrow cavities due to increased endosteal bone resorption, consisted with our previous report [21]. As expected, the values of BV/TV, Tb.N and Tb.Th in OVX femoral heads were significantly lower than those in Sham ones, the value of Tb.Sp in OVX rats was much higher, and the cortical thickness of femoral shaft was largely reduced after ovariectomy (P<0.001). (Figure **1 A-E**)

Primary MSCs isolated from osteoporotic rats were confluent after 12 d of culture, while the confluent time of MSCs from their sham littermate was 9d. To quantify the formation of extracellular mineralized nodules, Alizarin red staining was performed after 14 d in osteogenic medium. Nodule formation can be observed in both group, but the level of matrix mineralization was significantly lower in O-MSCs group (P<0.001). (Figure **1 F-J**) These results indicated that the osteogenic potential of MSCs was reduced after overiectomy. 

### 2: Cell morphology, proliferation and ALP activity on scaffolds

**Figure 2:**
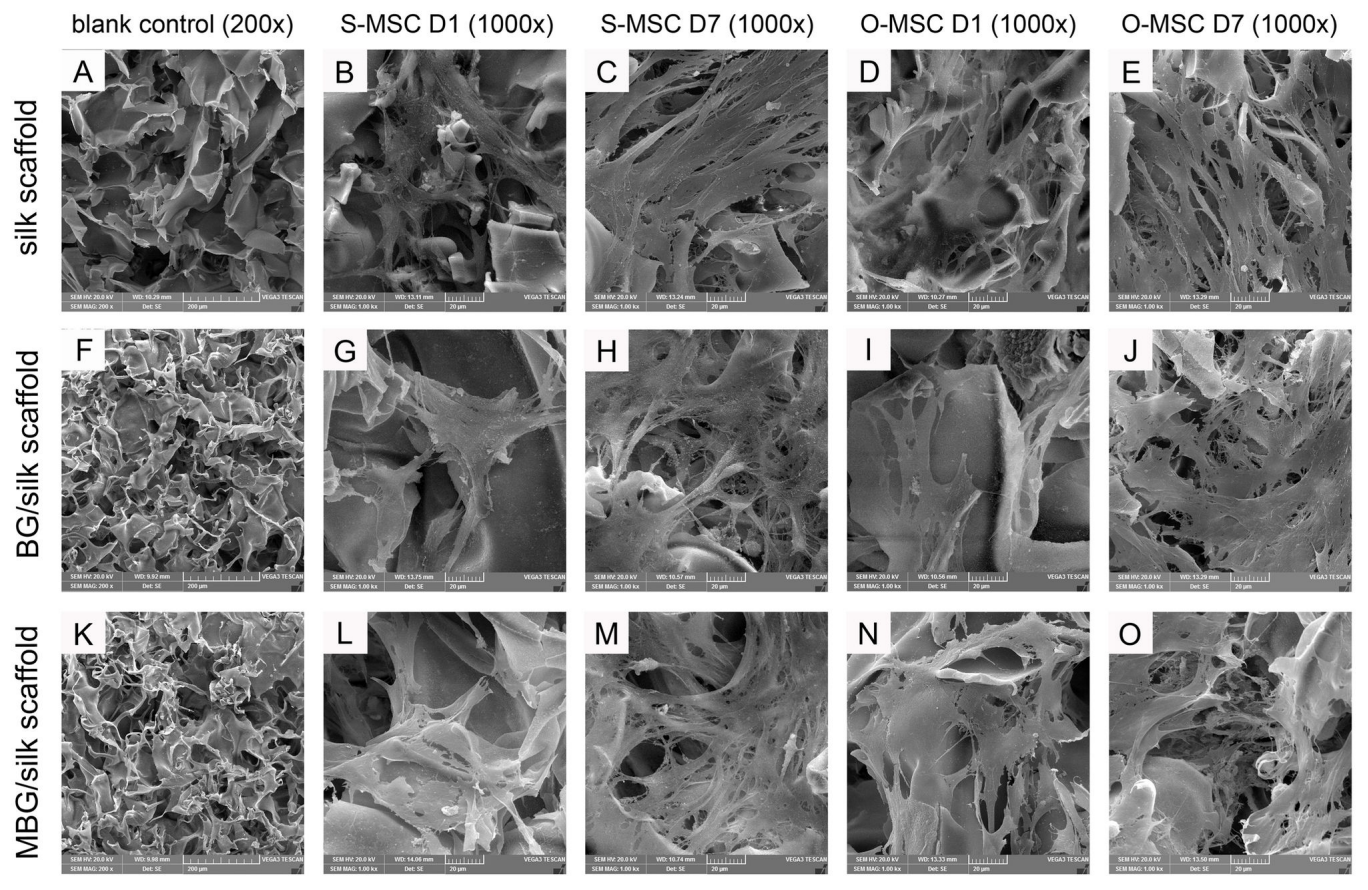
SEM images of cell seeded scaffolds. Blank control of silk (A), BG/silk (F) and MBG/silk (K) scaffolds in lower magnification (200x), and S-MSCs and O-MSCs cultured in the three scaffolds at day 1 and day 7 in higher magnification (B-E, G-J, L-O; 1000x). (Bar=200µm).

After 1 day culture, a small number of O-MSCs/S-MSCs were found to attach on the inner pores of all scaffolds. (Figure **2 B, G, L, D, I, N**) Then the number of cells increased gradually with spread out morphology, and cells contact each other with numerous cellular protrusions at day 7. O-MSCs displayed a flat appearance when attached to the pore surface, compared to S-MSCs. Both cells were uniformly distributed within all porous scaffolds, resembling a spider network. (Figure **2 C, H, M, E, J, O**)

**Figure 3:**
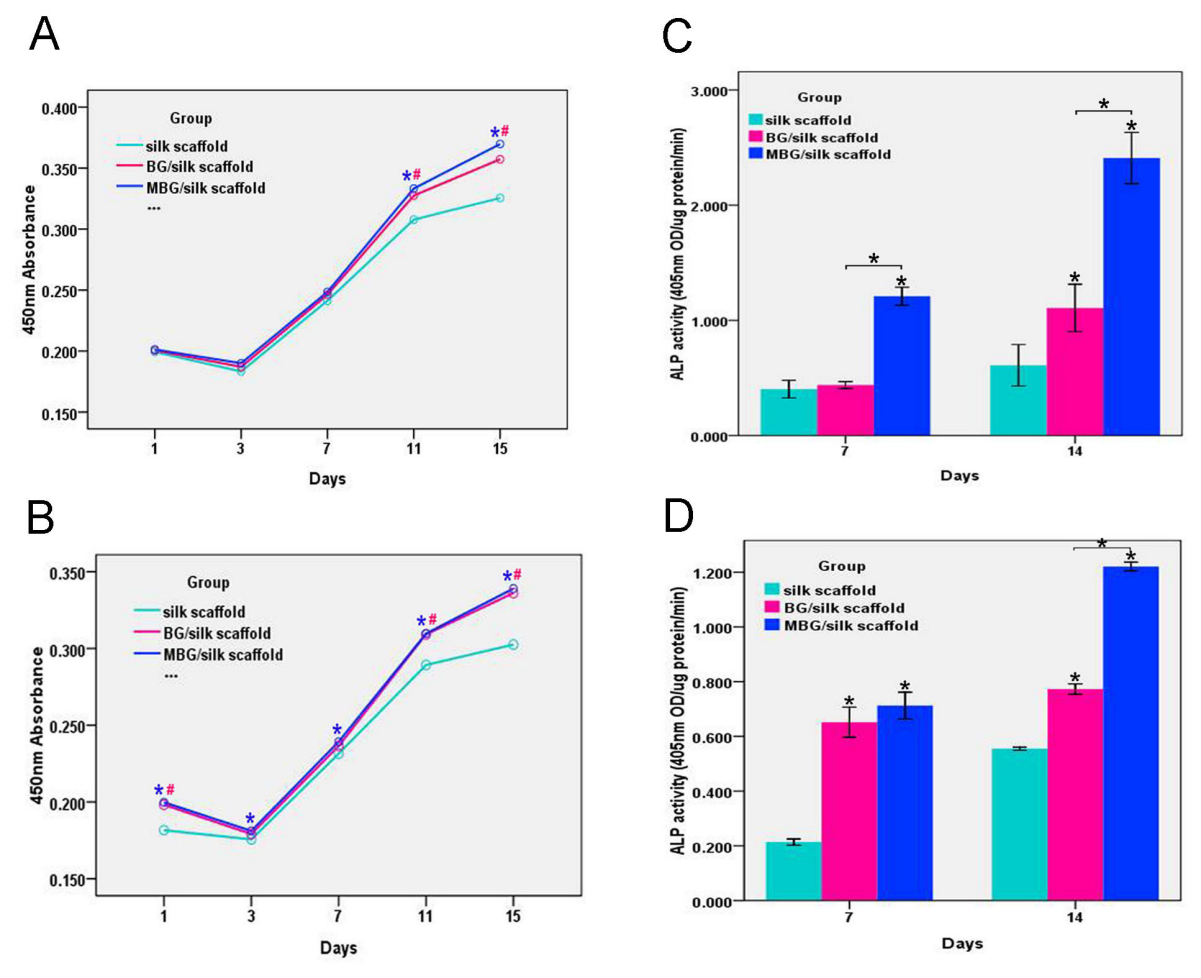
Cell proliferation and osteogenic differentiation. S-MSCs (A) and O-MSCs (B) proliferation in silk, BG/silk and MBG/silk scaffolds by CCK-8 assay at 1,3,7,11 and 15 d; (n=4 per group; #: P<0.05 BG/silk vs silk group, *:P<0.05 MBG/silk vs silk group) Quantitative Alp activity of S-MSCs (C) and O-MSCs (D) on silk, BG/silk and MBG/silk scaffolds at 7 and 14 d in osteogenic culture. (n=3 per group; *P<0.05).

To assess the cytocompatibility and viability of O-MSCs/S-MSCs on silk-based scaffolds, CCK-8 assay was performed to observe their growth profiles during 15 d in culture. Both BG/silk and MBG/silk scaffolds showed comparable growth pattern, while the pure silk showed a lower growth over time. (Figure **3 A, B**) At day 3, the slight reduction in proliferation curve of O-MSCs/S-MSCs in all groups indicate the decreased cell viability during incubation period, which was probably due to the temporary growth inhibition and limited nutrient supply within microstructure. Then, a steady increase of cell proliferation can be observed in the later time points. Cells performed higher proliferation in MBG/silk group at each time point compared with silk control (P<0.05), and reached a maximum value at day 15. This result reflects that these scaffolds were nontoxic for both O-MSCs and S-MSCs, and cells grew normally within the scaffolds.

The O-MSCs/S-MSCs were cultured on silk-based scaffolds in the presence of osteogenic medium to quantify ALP activity as an early marker of osteogenic differentiation. The ALP activity grown on all scaffolds increased from day 7 to day 14, and the highest level of ALP activity was detected for both cells grown on MBG/silk scaffold at both time points. (Figure **3 C, D**) For O-MSCs, no significant differences was detected between BG/silk or MBG/silk scaffolds at day 7, however, at day 14, O-MSCs grown on MBG/silk scaffold displayed a remarkably greater ALP activity than those on BG/silk and silk scaffolds (P<0.05). (Figure **3 D**)

### 3: New bone formation and mineralization within defects

**Figure 4:**
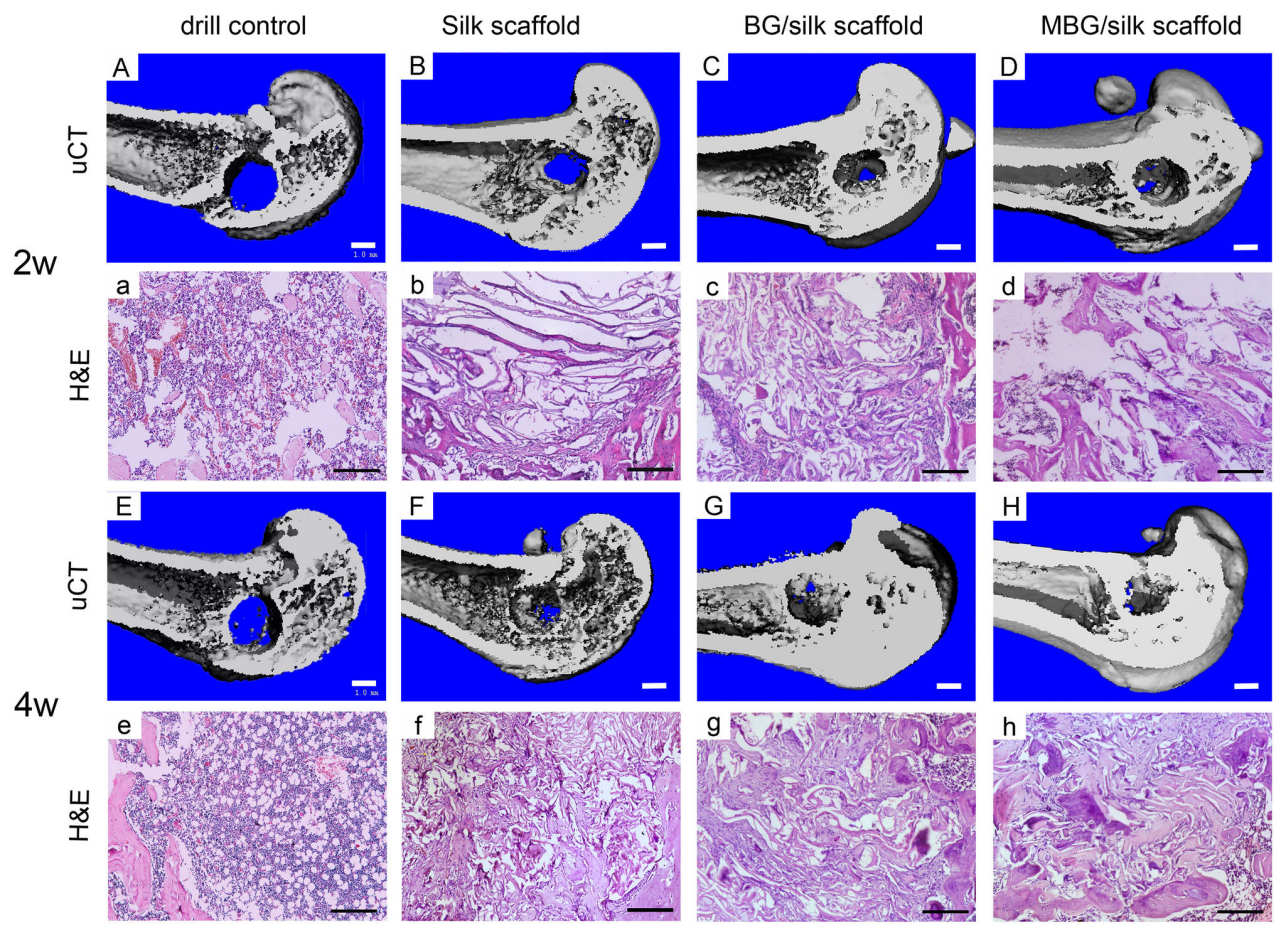
3D reconstruction of mineralized bone formation in the drill control, silk, BG/silk and MBG/silk groups at 2 and 4 weeks. (A-H; Bar=1mm) H&E staining of new bone matrix deposition within defects in the drill control, silk, BG/silk and MBG/silk groups. (a-h; Bar=200µm).

**Figure 5:**
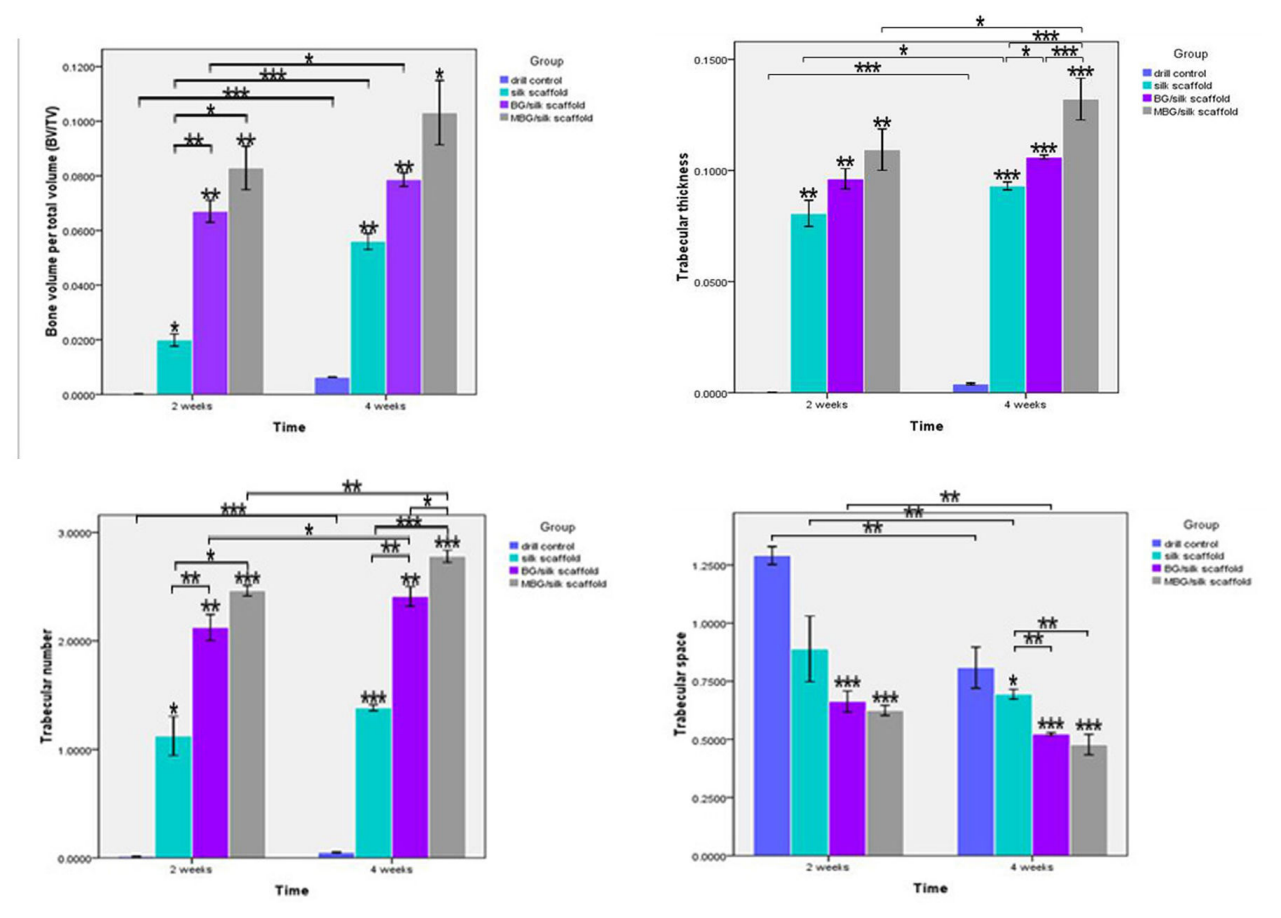
Quantitative data of BV/TV, Tb.Th, Tb.N and Tb. Sp within defects among the drill control, silk, BG/silk and MBG/silk groups at 2 and 4 weeks. (n=3 per group; * P<0.05, ** P<0.01, ***P<0.001).

For quantitative analysis of the bone morphology and mineralization, 3D reconstruction of μCT images was performed to detect new bone formation within defect. The analysis showed that drill-control defects failed to regenerate appreciable bone tissue during 4 weeks healing, indicating the critical size defects in osteoporotic femur failed to heal spontaneously. Only a small quantity of mineralized tissue without complete bridging was visible in the defect sites implanted with silk and BG/silk scaffolds at 2 weeks, whereas plate-like bone structures presented after 4 weeks transplantation. In contrast, MBG/silk scaffolds stimulated significantly more bone formation of plate-like pattern located in the center of the defect site, which almost expanded to fill the femur defect by 4 weeks. (Figure **4 A-H**) As indicators of the relative quantity of new bone formation, morphological parameters such as BV/TV, Tb.Th, Tb.N and Tb.Sp were used to detect mineralized bone tissue with defects. BV/TV (0.633%), Tb.Th (0.004 mm) and Tb.N (0.051 mm-1) were significantly low and Tb.Sp (0.809 mm) was relatively high in drill-control group by the end of the observation period, indicating minimal bone formation in drilled hole without treatment. At 2 weeks, BG/silk scaffolds showed a remarkable therapeutic effect in regenerating bone formation, with a significantly increase in BV/TV (3.4-fold), Tb.Th (19.3%) and Tb.N (89%), but a markedly decrease in Tb.Sp (25.4%) compared to pure silk. Meanwhile, values of BV/TV (8.3%), Tb.Th (0.109 mm) and Tb.N (2. 463 mm-1) of MBG/silk scaffolds were superior to that of BG/silk scaffolds. At 4 weeks, the MBG/silk scaffold group exhibited the highest value in BV/TV (10.3%), Tb.Th (0.132 mm) and Tb.N (2.78 mm-1), and 31.2% less in Tb.Sp compared with silk control. (Figure **5**)

**Figure 6:**
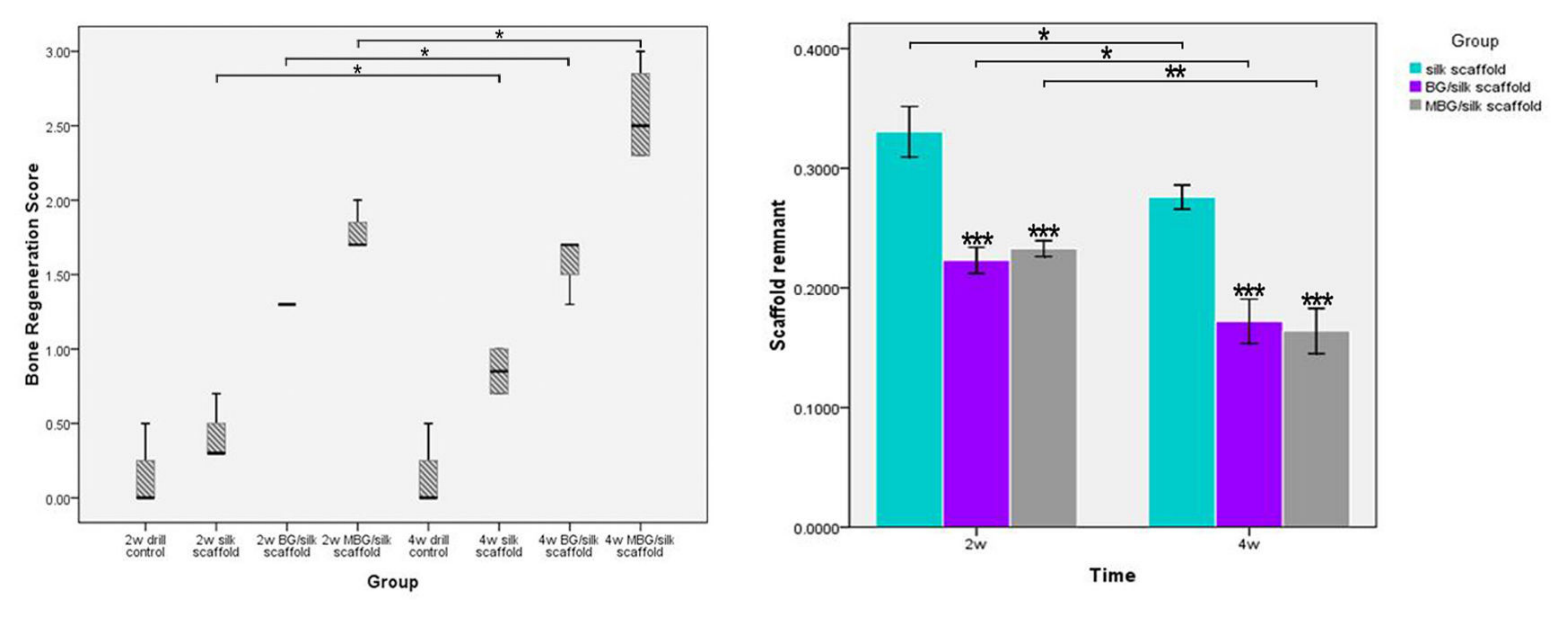
Bone regeneration and scaffold remnant within defect. (A) Semi-quantitative scores of bone regeneration in the femur defects are presented as box plots, where the boxes represent the first and third quartiles. (B) Quantitative data of scaffold remnant fraction in BG/silk and MBG/silk group at 2 and 4 weeks. Four sections per sample in each group were used at each time point per analysis. Scaffold remnant=area of silk scaffolds/total area; * P<0.05, **P<0.01, ***P<0.001.

**Figure 7:**
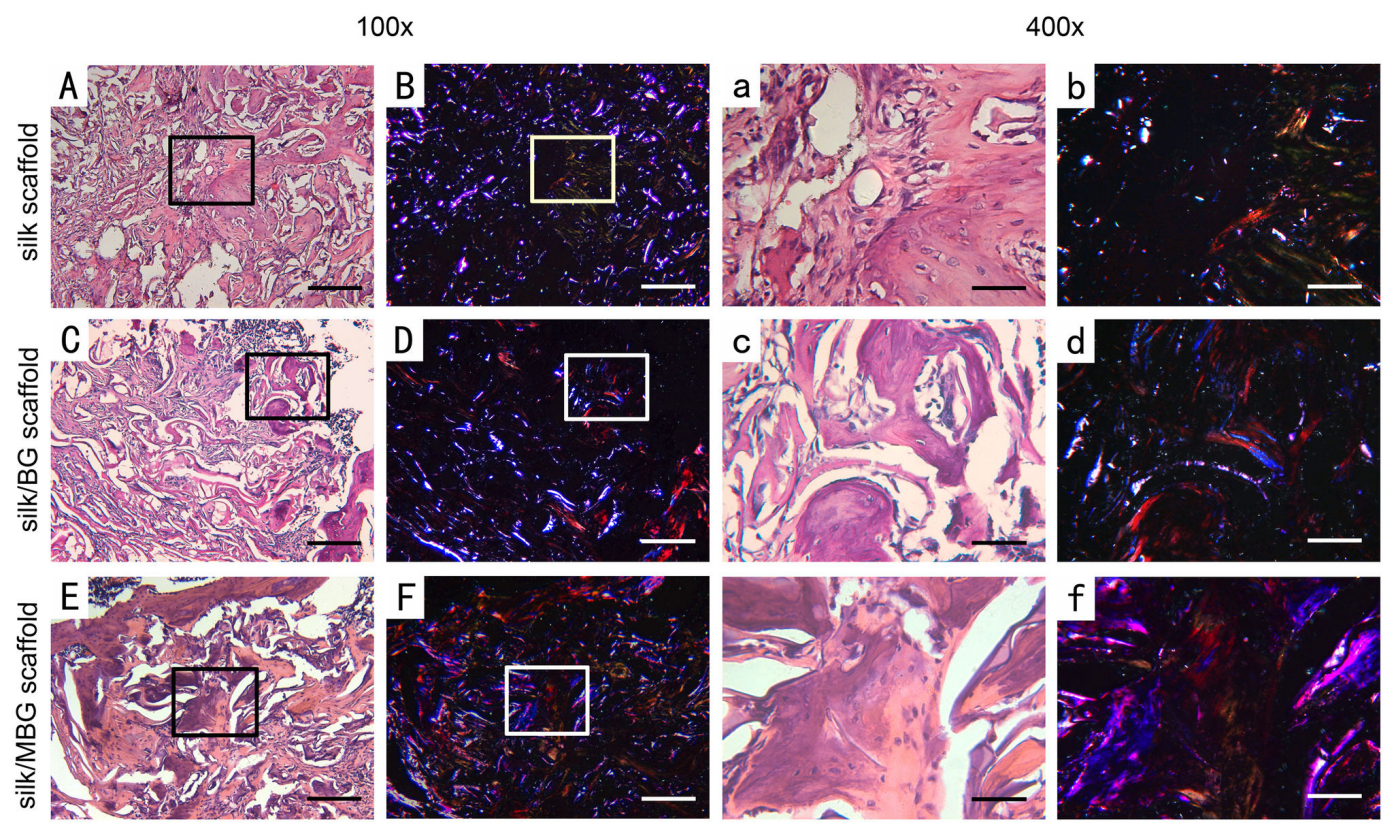
Polarized light micrographs. Images of in vivo bone formation in the silk (B,b), BG/silk (D,d) and MBG/silk (F,f) groups at 4 weeks, with the same field of silk (A,a), BG/silk (C,c) and MBG/silk (E,e) groups shown in H&E staining. Scaffolds are bright white in dark field, and mature collagen fibers are red or yellow. More mature collagen matrix appears in MBG/silk group. Lower magnification (x100; A-F; bar=200 µm); higher magnification (x400; a-f; Bar=50µm).

**Figure 8:**
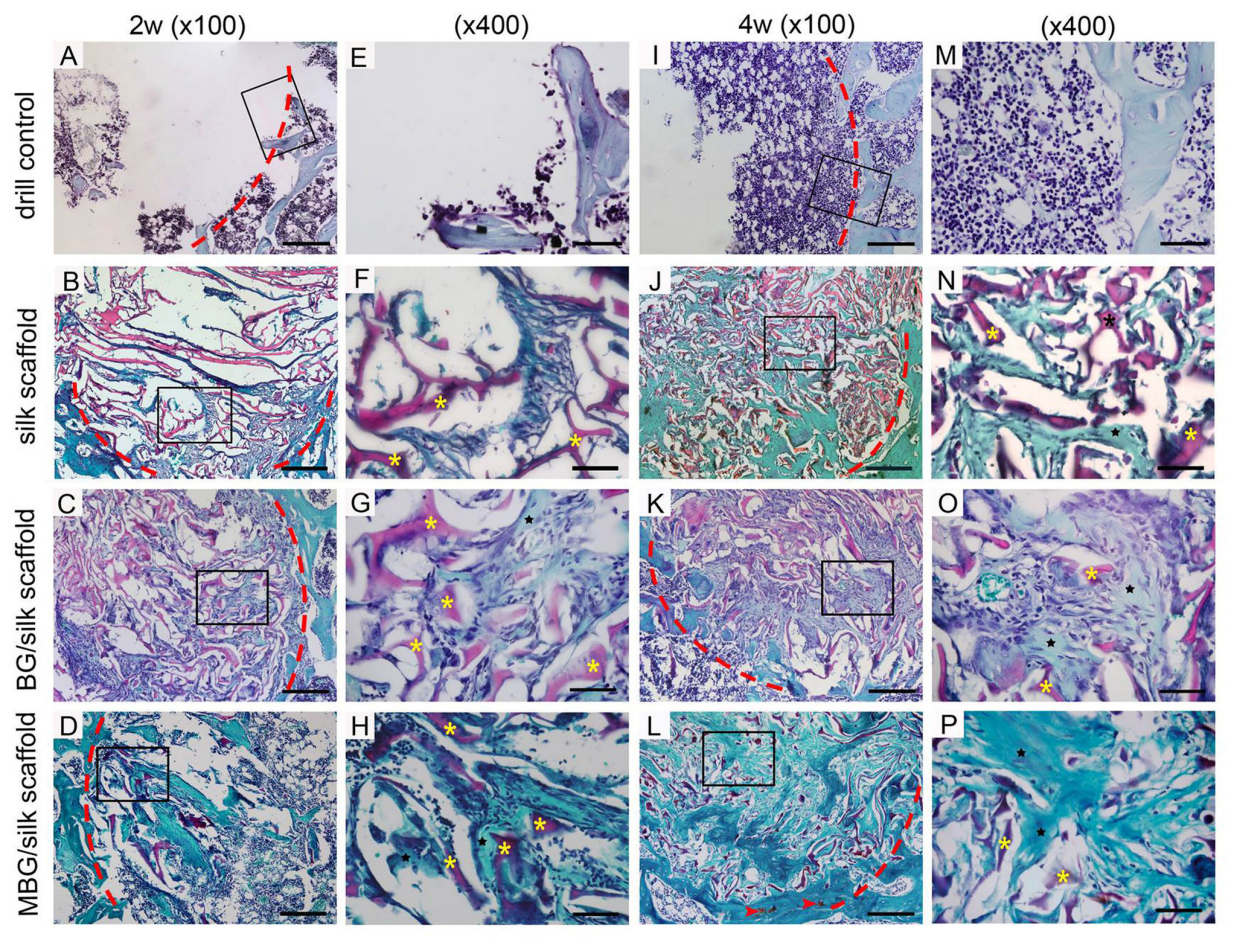
Saffranin O staining of bone formation (black star) and scaffolds remnant (yellow star) within defects in the drill control, silk, BG/silk and MBG/silk groups at 2 and 4 weeks. Traces of cartilage matrix (red arrow head) can be observed in MBG/silk groups. Lower magnification (x100; A-C, I-L; bar=200 µm); higher magnification (x400; E-H, M-P; Bar=50µm). The red dotted line indicated defect margin.

Histomorphometric evidence of bone matrix deposition further supported the findings obtained by μCT analysis. In the drill-control defects, abundant bone marrow-like tissue and oval vacuolar adipocytes with a clear boundary of host bone can obviously be seen within drill-control defect at both time points, but poorest bone formation was presented (P<0.05). (Figure **4 a-h**; Figure **6 A**) Substantial novel bone tissue could be observed in MBG/silk scaffold group, leaving a disappearing boundary between host bone and regenerated bone tissue. Unlike the microstructural deteriorated host bone, the regenerated woven bone was well deposited and structured both at the periphery and in the center of defect sites during the whole healing period. The MBG/silk scaffolds failed to maintain their structure integrity and markedly degraded into small fragments, making way for the increasing amount of osteogenic cells and osteoid by 2 weeks and more maturated bone tissue at 4 weeks in defect center (P<0.05). It indicated the most rapid degradation rate of MBG/silk in this model. Polarized light micrographs revealed more mature fibrous bone tissue at 4 weeks in MBG/silk group, compared with silk and BG/silk group. (Figure **7**) The majority of the bone tissue was located at the defect margin in BG/silk scaffolds, with increased quantity of osteoblastic cells and osteoid and some few capillaries randomly dispersed through remnant scaffolds that were less degraded. The appearance of remaining scaffolds (P<0.05) and pore structural integrity of pure silk was better maintained with less bony ingrowth and higher quantity of centrally located connective tissue, compared with BG/silk and MBG/silk scaffolds (P<0.01). (Figure **8**; Figure **6 B**)

Noticeably, in BG/silk scaffolds, the newly formed border bone was quite histologically similar to the surrounding disorganized osteoporotic bone at 2 weeks, however, at 4 weeks, the bone structure improved and turned to be sound trabeculea-like tissue. By contrast, at each time point, the morphology of regenerated bone tissue from MBG/silk scaffolds group was constantly plate-like and well organized at the periphery, accompanied with traces of cartilage matrix, suggesting an activated remodeling process both through intramembranous and endochondral bone formation. (Figure **8**) 

### 4: Immunohistochemical assessment

**Figure 9:**
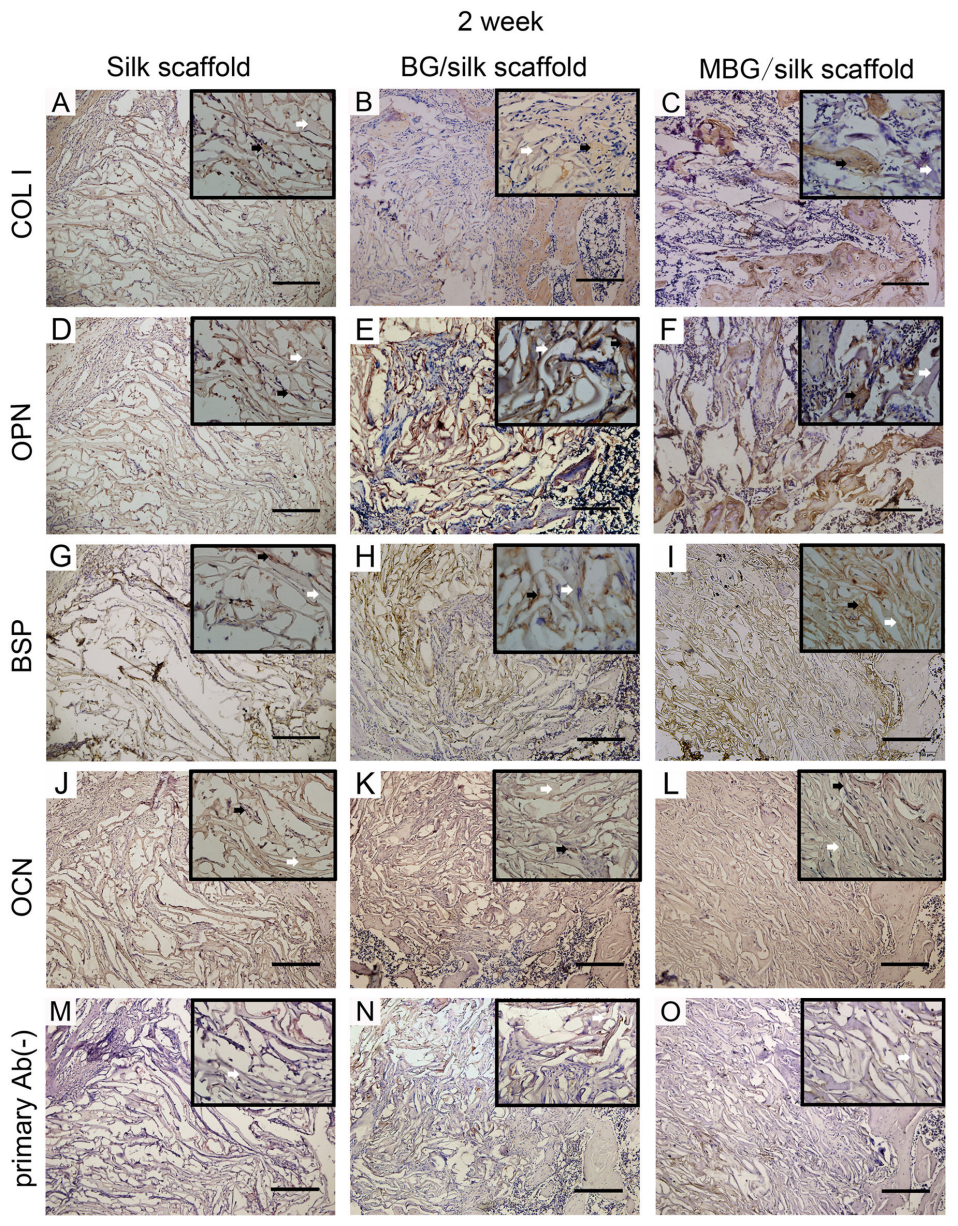
Immunohistochemical markers of COL I, OPN, BSP, OCN and primary Ab(-) control in silk, BG/silk and MBG/silk groups at 2 weeks. Black arrow indicated the positive staining in bone forming tissue, and white arrow indicated silk scaffold. (Bar=200µm).

**Figure 10:**
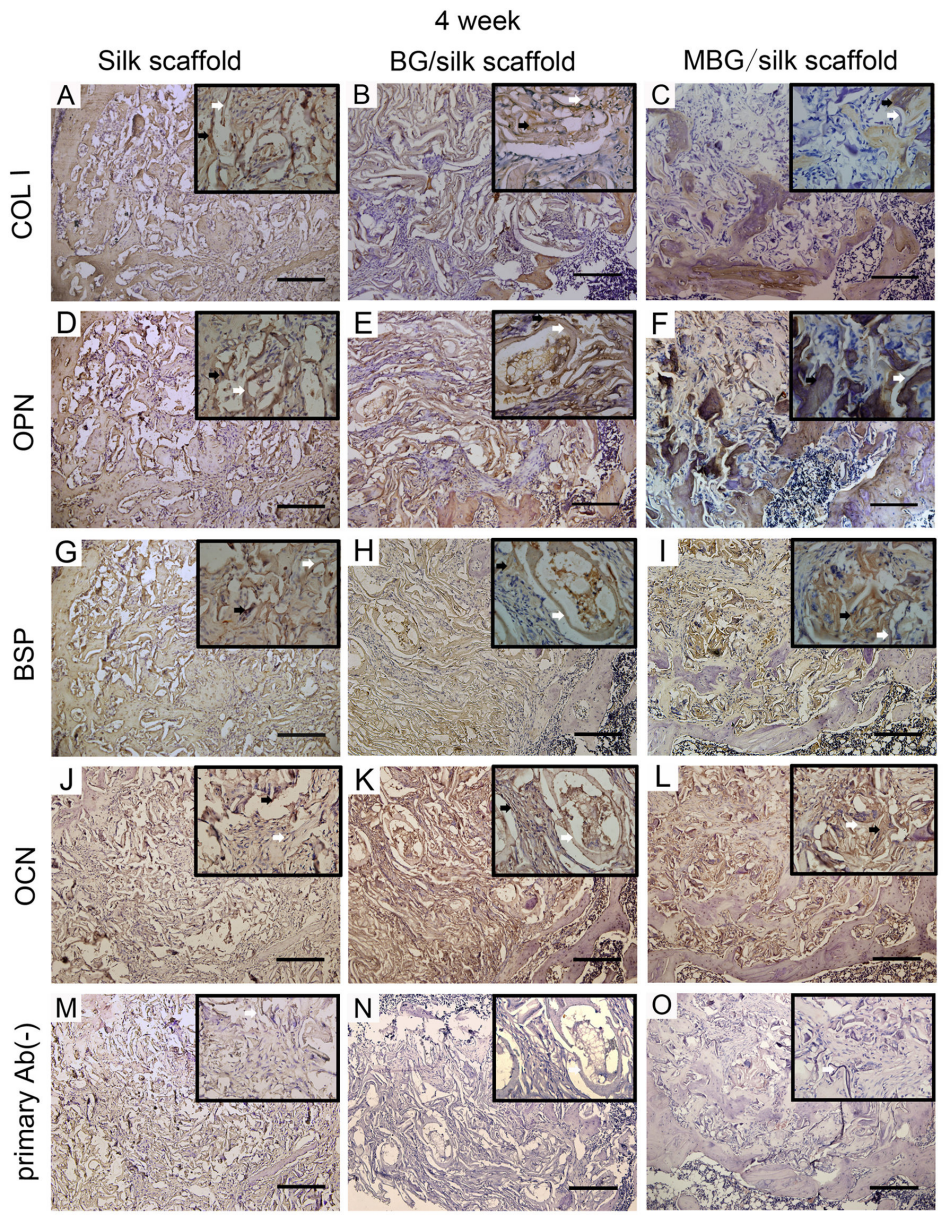
Immunohistochemical markers of COL I, OPN, BSP, OCN and primary Ab(-) control in silk, BG/silk and MBG/silk groups at 4 weeks. Black arrow indicated the positive staining in bone forming tissue, and white arrow indicated silk scaffold. (Bar=200µm).

In addition to the distinguishing deposition of mineral, the production of bone matrix proteins, such as COL1, OPN, BSP and OCN were detected over time by immunohistochemical staining among all transplanted scaffolds. (Figure **9**; Figure **10**) Noticeably, the staining of four proteins had significantly greater intensity and area in MBG/silk scaffolds than silk and BG/silk scaffolds. The intensity of positive staining increased with time and peaked at 4 weeks of observation, especially located peripheral to the newly formed border bone. (Figure **10**) The results indicated that MBG/silk scaffolds can induce accelerated collagen and non-collagen matrix synthesis and deposition in both the initial and late phase of bone regeneration. The slight positive staining in primary Ab(-) sections was probably due to non-specific binding to silk fibroin.

**Figure 11:**
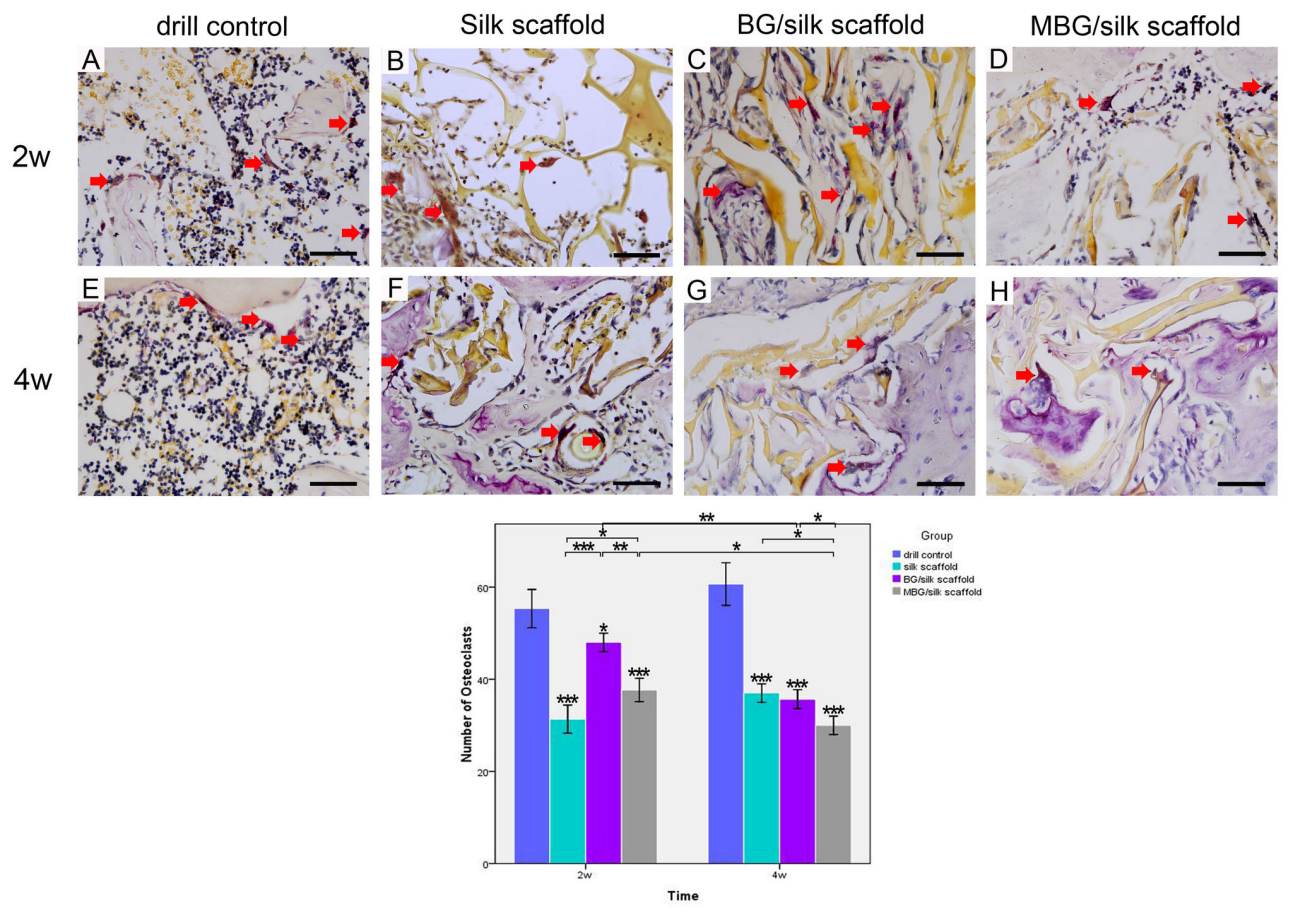
TRAP staining within defects in the drill control, BG/silk and MBG/silk groups at 2 and 4 weeks. (A-H; Bar=50µm) The number of TRAP-positive osteoclasts was counted at low magnification (x100). * P<0.05, ** P<0.01, ***P<0.001.

Representative TRAP staining exhibited an increasing amount of mononuclear macrophages in drill-controls, indicating an imbalance status towards bone resorption in osteoporotic defects. At 2 weeks, mature osteoclasts (nuclei of TRAP positive staining cells≥3) fused by mononuclear macrophages could be seen surrounding remnant scaffolds in all silk-based scaffold groups. Meanwhile, more TRAP positive cells co-existed with osteoblastic cells were found in BG/silk scaffold group, leading to an activated bone remodeling process. Both BG/silk and MBG/silk scaffolds exhibited a tendency of reduced osteoclast number during 4 weeks healing. The smallest quantity of TRAP-positive cells with mature morphology were randomly scattered within MBG/silk scaffolds at the end of observation (P<0.001), revealing a milder inflammatory response provoked by the former one. (Figure **11**)

## Discussion

Tissue engineering-based approach represents a promising alternative for traditional osteoporosis therapies, especially for osteoporotic defect repair, based on the optimal advantages of 3D porous scaffolds, such as providing an interconnected macroporous network to allow cell migration, nutrient delivery, bone ingrowth and vascularization. Although good biocompatibility and osteoconductivity of silk-based scaffolds has been found both *in vitro* and *in vivo* in normal bone tissue, study of composite MBG/silk scaffolds on skeletal metabolic disease condition, such as osteoporotic, was scant. Thus, the results of our study showed that 10 wt. % MBG/silk 3D scaffolds could enhance attachment, proliferation and ALP activity of both normal and ovariectomized MSCs, and accelerate bone formation with compatible scaffold degradation and reduced osteoclastic response of defect healing in OVX rats compared to silk and BG/silk scaffolds, which approved our prior hypothesis. 

BGs were first discovered by Hench et al. [28] with a SiO_2_-CaO-P_2_O_5_ composition and now considered as the bioactive and resorbable material for biomedical applications. Recent research has been focusing on the molecular interaction between ionic dissolution products of BGs with their physiological environment, so as to get a deeper understanding of the mechanisms and to specifically fabricate “smart” glasses with ideal properties for bone tissue engineering[6,29]. Their dissolution product of Si ion was demonstrated to promote osseointegration [30] and mineralization [31] in the initiation stage of bone tissue, as well as to facilitate angiogenesis investigated from both *in vitro* and *in vivo* studies[32]. Jugdaohsingh et al. [33] reported that dietary Si intake can increase the bone mineral density (BMD) both in men and premenopausal women. The concentration of extracellular Ca ion released from BGs also plays an important role in bone remodeling. Ca ion can directly activate intracellular mechanisms by affecting Ca-sensing receptors in osteoblastic cells, followed by increasing the expression of Insulin like growth factors IGF-I or IGF-II to regulate human osteoblast proliferation[34]. Besides, inorganic phosphate is another essential element for hydroxyapatite deposition and bone matrix mineralization. Wittrant et al. [35] recently found out that P has the effect of up-regulating Glvr-1 and Glvr-2 in murine odontoblast-like cells correlated with ERK1/2 phosphorylation and CaP crystals formation, which also requires the co-presence of Ca ion in these signalling pathways. The above evidences also prove the hypotheses postulated by Hench [36] that “ionic dissolution products released from bioactive glasses stimulate the genes of cells towards a pathway of regeneration and self-repair”. 

Noticeably, cell response to biomaterial depends not only on chemical composition, but also on surface area, topography and texture properties (pore size, pore volume) of the scaffolds[29,37]. MBGs were developed with the same composition of BGs but a highly ordered mesopore channel structure by a combination of the sol-gel method [10]. The increased specific surface area and pore volume of MBGs greatly accelerates the nano-sized hydroxyapatite (nHA) formation and its degradation products promote the bone tissue regeneration[38]. Clearly, it is generally believed that higher CaO content in BGs leads to faster apatite formation, which is absolutely different from the composition/bioactivity correlation of MBGs that the *in vitro* bioactivity depends on the Si/Ca ratio in the network when the other material parameters such as the mesostructure and texture properties are controlled[39]. Yan et al. [39] prepared ordered MBGs with different Si/Ca ratios and compared the *in vitro* bioactivity, the results demonstrated that the composition /bioactivity sequence is 80Si/15Ca70Si/25Ca60Si/35Ca95Si/5Ca100Si, indicating 80Si/15Ca has the best bioactivity. In our study, we use the same composition of 80Si/15Ca to investigate the osteogenic potential. Moreover, MBG has been reported to be successfully load with ipriflavone, an anti-osteoporotic drug, to achieve long-term delivery [40], indicating the therapeutic potential of MBG-based scaffolds in osteoporosis. 

Silk fibroin has been widely studied for bone and cartilage repair applications in the form of pure silk or its composite scaffolds[13,41]. Silk exhibits the advantages of water-soluble nature for non-cytotoxic scaffold preparation when compared to traditional polymer materials such as PLGA and PLLA[42], and superior mechanical properties to compensate for the brittleness of ceramics [11]. According to our previous study, silk modification could induce a more uniformly distributed pore network within the MBG scaffolds [11], and strengthen the scaffolds by linking the inorganic phase together[43]. The composite MBG/silk scaffolds exhibited excellent apatite-formation abilities, enhanced compressive strength similar to cancellous bone, faster dissolution rate, more stable PH environment and superior *in vivo* osteogenic ability compared to BG/silk scaffolds [11,20], suggesting a potential therapeutic efficacy for bone defect healing. 

Osteoporosis is caused by the imbalance activities between bone-forming osteoblasts and bone-degrading osteoclasts during remodelling process, at the cellular level. Extracellular pH value is known to play an important role to balance bone formation and resorption process[44]. A local alkalinized microenvironment might be important for bone regeneration in osteoporosis, since ALP activity towards inorganic pyrophosphate was increased at pH 8.5 and the osteoclastic resorption was accelerated in more acid environment[44,45]. In this study, only a small number of osteoclasts existed in MBG/silk scaffolds, while more mononucleate giant cells or mature osteoclasts can be observed in other groups. This can be explained by the raised PH environment through robust release of Si ion from MBG. The results are consistant with another *in vitro* finding that the presence of MBG maintains the high viability of human Saos-2 osteoblasts, murine L929 fibroblasts and murine SR.D10 T lymphocytes without modifying *in vitro* T-cell response, suggesting its excellent biocompatibility for bone and dental application[46]. 

Some likely factors may also explain this superior therapeutic efficacy: First, the highly ordered channel structure of MBG provides a suitable space for local bone remodeling initiated by stimulating the proliferation of osteoblasts and recruiting osteoprogenitors to defect location. Second, the degradation rate of MBG/silk scaffold perfectly matches the bone formation rate at this defect site, indicating the degraded scaffolds make way for newly secreted bone matrix and sequential mineralization. Third, since the pore size and pore structure play an important role in the protein-adsorption behavior for mesoporous materials [47], MBGs with a controllable pore size and mesostructure may significantly enhanced the ability of protein adsorption and influence nutrient delivery for tissue regeneration. Fourth, MBG has the ability to bind spontaneously with bony tissue through the formation of nHA layer. The faster dissolution rate of MBG/silk scaffolds compared to BG/silk scaffolds may enhance bone formation; this is supported by the evidences that the products of released Si ions at the interface could form Si(OH)_4_ which not only stimulate COL I synthesis [48] but also reverse acid microenvironment in osteoclastic imbalance to provide a more stable PH environment[49]. The important role of Si is also proved by showing that CaSiO_3_ ceramics degrades significantly faster than β-tricalcium phosphate ceramics and leads to an improved *in vivo* osseointegration[30]. 

## Conclusion

In conclusion, this study suggests that synthesized MBG/silk scaffolds can be used as bone substitutes for local implantation into critical sized osteoporotic defects, owing to the enhanced *in vitro* cell attachment, proliferation and osteogenic differentiation, and accelerated *in vivo* healing progress over skeletal deterioration compared with silk and BG/silk scaffolds. The rapid mineralization and reduced osteoclastic activity of MBG/silk scaffolds suggests their potential therapeutic efficacy especially in the site of post-menopausal osteoporosis.
